# Panax Notoginseng Saponins Prevent Bone Loss by Promoting Angiogenesis in an Osteoporotic Mouse Model

**DOI:** 10.1155/2020/8412468

**Published:** 2020-12-14

**Authors:** Hao Hu, Yan Chen, Zhiyuan Zou, Liangping Li, Fuxin Wei, Chun Liu, Zemin Ling, Xuenong Zou

**Affiliations:** ^1^Guangdong Provincial Key Laboratory of Orthopedics and Traumatology, Department of Spinal Surgery, The First Affiliated Hospital of Sun Yat-sen University, Guangzhou 510080, China; ^2^Department of Orthopaedic Surgery, The Seventh Affiliated Hospital and Orthopedic Research Institute of Sun Yat-sen University, Shenzhen 518107, China; ^3^Precision Medicine Institute, The First Affiliated Hospital of Sun Yat-sen University, Guangzhou 510080, China

## Abstract

With the aging of the population and the extension of life expectancy, osteoporosis is becoming a global epidemic. Although there are several drugs used to treat osteoporosis in clinical practice, such as parathyroid hormone or bisphosphonates, they all have some serious side effects. Therefore, a safer drug is called for osteoporosis, especially for the prevention in the early stage of the disease, not only the treatment in the later stage. Panax notoginseng saponin (PNS), a traditional Chinese herb, has been used as anti-ischemic drug due to its function on improving vascular circulation. In order to verify whether Panax notoginseng saponins (PNS) could be used to prevent osteoporosis, ovariectomy (OVX) was induced in female C57BL/C6J mice, followed by orally administration with 40 mg/kg/d, 80 mg/kg/d, and 160 mg/kg/d of three different dosages of PNS for 9 weeks. Serum biochemical analysis, micro-CT, histological evaluation, and immunostaining of markers of osteogenesis and angiogenesis were performed in the sham, osteoporotic (OVX), and treatment (OVX+PNS) groups. Micro-CT and histological evaluation showed that compared to sham group, the bone mass of OVX group reduced significantly, while it was significantly restored in the moderate-dose PNS (40 mg/kg and 80 mg/kg) treatment groups. The expression of CD31 and osteocalcin (OCN) in the bone tissue of treatment group also increased, suggesting that PNS activated osteogenesis and angiogenesis, which subsequently increased the bone mass. These results confirmed the potential function of PNS on the prevention of osteoporosis. However, in the high dose of PNS (160 mg/kg) group, the antiosteoportic effect had been eliminated, which also suggested the importance of proper dose of PNS for the prevention and treatment of osteoporosis in postmenopausal women.

## 1. Introduction

Osteoporosis is a metabolic disease characterized by decreased bone mass, increased bone fragility, and microarchitectural deterioration of bone tissue. In addition to increasing the risk of fragility fracture, osteoporosis may also increase the risk of hospitalization associated with certain complications. With the increasing aging of China's population, the prevalence of osteoporosis has increased remarkably in the past decade (from 14.94% before 2012 to 27.96% in 2015), and the prevalence in women was significantly higher than that in men (25.41% vs. 15.33%) [1].

With aging, decreased angiogenesis in bone and bone marrow is a principal cause of osteoporosis [2]. Because estrogen is also an important regulator for angiogenesis, its deficiency can lead to osteoporosis for postmenopausal women or those who have undergone tumor-related ovariectomy. Traditional Chinese medicine has been used for thousands of years and still plays an important role in the prevention and the treatment of diseases in modern China. The dried root of Panax notoginseng, namely, Sanchi (San Qi), is a famous traditional Chinese herb. The active ingredients of Panax notoginseng include saponins, dencichine, flavonoids, and polysaccharides, among which the pharmaceutical effects of the saponins had been studied extensively [3]. The total saponins of Panax notoginseng mainly include ginsenosides Ra3, Rg1, Rb1, and Rd and notoginsenoside R1. Blood concentration of ginsenosides Ra3, Rb1, and Rd was significantly higher than other compounds after an oral administration of Panax notoginseng extract in a rat model [4]. Panax notoginseng saponin (PNS) is a vasoactive drug [5], which has been used as an anti-ischemic agent to promote blood circulation in traditional Chinese medicine; however, it also displayed anti-inflammatory [6, 7], antioxidant [8–11], and estrogen-like [12, 13] activities in vitro, making it a potential cure for postmenopausal osteoporosis.

Some previous studies confirmed that Panax notoginseng saponins facilitate the osteogenic process of the skeletal progenitor cells in vitro [14, 15], including the proliferation, differentiation, and mineralization. The possible mechanism is that PNS promotes the expression of downstream osteogenesis-related genes by activating ERK and p38 [16] as well as TGF-*β*1 [17] signaling pathways. Angiogenesis plays an indispensable role in osteogenesis [18], and the most important pharmacological function of PNS is its vasoactive effect, indicating that PNS could preserve bone mass by promoting the coupling of angiogenesis and osteogenesis during osteoporosis. Although other two studies had also proved the pharmacological effect of PNS on alleviating osteoporosis in rat ovariectomy model [19, 20], the underlying mechanisms were not investigated, especially there were no studies to evaluate the function of PNS on preventing bone loss at the early stage of menopause. In this study, we established an ovariectomy-induced osteoporosis mouse model to fully investigate whether early PNS treatment can prevent bone loss by targeting the vascular microarchitecture.

## 2. Materials and Methods

### 2.1. Materials

PNSs, the total saponins of Panax notoginseng, were purchased from KPC Xuesaitong Pharmaceutical Co., Ltd (Yunnan, China). C57BL/6J mice were purchased from SPF (Beijing) Biotechnology Co., Ltd (Beijing, China). Mouse NTXI(cross linked N-telopeptide of type I collagen) ELISA kit (E-EL-M3022) was purchased from Elabscience (Wuhan, China). Anti-osteocalcin antibody (ab93876), recombinant anti-CD31 antibody (ab182981), goat anti-rabbit IgG H&L (Alexa Fluor® 488) (ab150077), and goat anti-rabbit IgG H&L (HRP) (ab205718) were purchased from Abcam (USA).

### 2.2. Animal Model and PNS Treatment

All the animal experiments were carried out carefully in accordance with the principles and guidelines of the Animal Ethics Committee of the First Affiliated Hospital of Sun Yet-sen University. 30 twelve-week-old female C57BL/6J mice were housed in individual ventilated cages under controlled conditions (temperature, 20-26°C; humidity, 40-70%) for a 12-hour light-dark cycle and were allowed free access to water and food.

After one-week adaptation period, the animals were randomly divided into 5 equal groups (six in one cage): (1) sham operation (sham group), (2) ovariectomy (OVX group), (3) ovariectomy+40 mg/kg/d PNS (low-dose group), (4) ovariectomy+80 mg/kg/d PNS (medium-dose group), and (5) ovariectomy+160 mg/kg/d PNS (high-dose group). Ovariectomy or a sham operation was performed under pentobarbital sodium (90 mg/kg, i.p.) anesthesia. Two longitudinal incisions were made inferior to the rib cage on the dorsolateral body wall, and then the bilateral ovaries were exteriorized, ligated, and excised. Mice in the sham surgical group had only a piece of fat excised. After the surgery, mice in PNS-treated groups were orally administered (oral gavage) with different dosages for 9 weeks, while mice in the sham group and the OVX group were also orally administered with water in the same volume. After 9-week treatment, all mice were euthanized. Eyeball blood collection was conducted, and serum was separated by centrifugation at 1,800 rpm for 10 minutes, then were aliquoted and stored at -20°C for further analysis. PBS and then paraformaldehyde were perfused into the whole body through the left ventricle. Femora were dissected and stored at 4% paraformaldehyde at 4°C for further analysis.

### 2.3. Serum Biochemical Analysis

Serum samples were sent to Kingmed Diagnostic (Group Co., Ltd) for conventional biochemical analysis, including serum alanine aminotransferase (ALT), creatinine (CREA), serum calcium (S-Ca), and serum phosphorus (S-P). Serum bone turnover markers like N-telopeptide of type I collagen (NTX) were tested by using a commercial ELISA kit (E-EL-M3022). Serum ALT and CREA were tested to verify whether PNS reveals a dose-dependent hepatorenal toxicity. S-Ca, S-P, and NTX were tested for bone metabolism.

### 2.4. Micro-CT Analysis

The microarchitectures of the distal femurs were analyzed by a desktop Micro-CT SkyScan1276 (Bruker Micro CT, Belgium). In our work, micro-CT scanner was operated at 85 kV and 200 *μ*A. 1 mm thickness aluminium filter was used for optimal image contrast. Images were reconstructed and processed with a spatial scanning resolution of 10.0 *μ*m. Software CTAn (Bruker micro-CT, Belgium) was used to perform image analysis. Trabecular bone was separated from cortical bone by free-drawing region of interest (ROI). Volume of interest (VOI, 1 mm proximal to the metaphyseal line) was chosen within 100 continuous slices. We performed bone morphologic measurements in CTAn and obtained corresponding parameters, including trabecular bone volume fraction (BV/TV; %), trabecular thickness (Tb.Th; *μ*m), trabecular number (Tb.N; *μ*m^−1^), and trabecular separation (Tb.Sp; *μ*m). Then, the 3D models of VOI were reconstructed with CTAn for visualization in the software CTVol (Bruker micro-CT, Belgium). The operator conducting the micro-CT analysis was blinded to the treatments associated with samples.

### 2.5. Histological Assessment and Immunostaining

After micro-CT analysis, all femur specimens were prefixed at 4% paraformaldehyde for 48 h, decalcified in 10% EDTA (pH 7.4) for 21 d at 4°C, and then embedded in paraffin. We processed 4 *μ*m thick sagittal-oriented (longitudinally) sections of bone including the metaphysis and diaphysis. All slides were stored at 4°C in case of any further analysis. HE staining and Safranin O-Fast Green staining were performed for the analysis of bone microstructure.

Immunohistochemistry and immunofluorescence staining were applied to analyze osteogenesis and angiogenesis according to standard protocols. The sections were incubated at 4°C overnight with primary antibodies anti-osteocalcin (ab182981, Abcam, 1 : 500) and anti-CD31 (ab93876, Abcam, 1 : 500), respectively; the corresponding secondary antibodies were added onto the sections for 1 h. For osteocalcin immunohistochemistry, slides were stained with DAB (ab64238, Abcam) and then counterstained with hematoxylin (Sigma-Aldrich). For CD31 immunofluorescence, slides were counterstained with DAPI. The slide images were observed and captured by Eclipse Ti-SR microscope (Nikon, Japan). ImageJ was used for the following quantitative analysis.

### 2.6. Statistical Analysis

Statistical analysis was performed by using the SPSS 22.0 software (IBM Corp., Armonk, NY, USA). All data were presented as mean ± S.D. All error bars in figures represent S.D. Group comparison was made by using unpaired, two-tailed Student's *t*-test. For all statistical analysis, ^∗^*P* < 0.05 was considered to be significant.

## 3. Results

### 3.1. Body Weight

**Figure 1:**
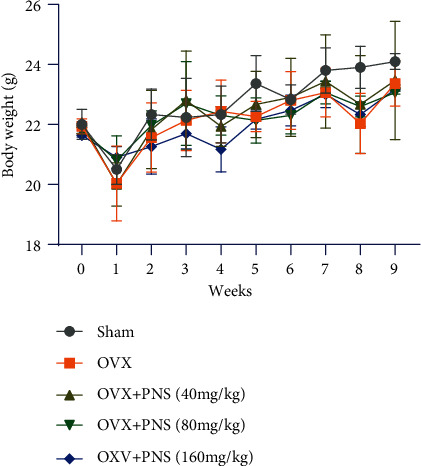
Plot of body weight of mice with respect to time, recorded over a period of pre- and 9 weeks postoperation. Values are presented as means ± S.D. (*N* = 6).

All the animals had normal activities and feeding during the whole experimental period. We monitored the body weight of mice in each group before operation and 1-9 weeks postoperation (Figure 1). Generally, the body weight of mice in all groups increased until they were sacrificed. Because of the surgical trauma, the body weight of each group decreased significantly (*P* < 0.05) one week right after operation. With the rehabilitation and growth of the mice, the body weight in each group gradually increased. There was no significant difference in body weight among the OVX and PNS-treated groups at each time point, indicating that the intake of PNS did not have a significant impact on body weight. Specially, the body weight of the sham operation group was significantly higher (*P* < 0.05) than that of the OVX group at the eighth week, which may due to less surgical trauma.

### 3.2. Serum Biochemical Analysis

**Figure 2:**
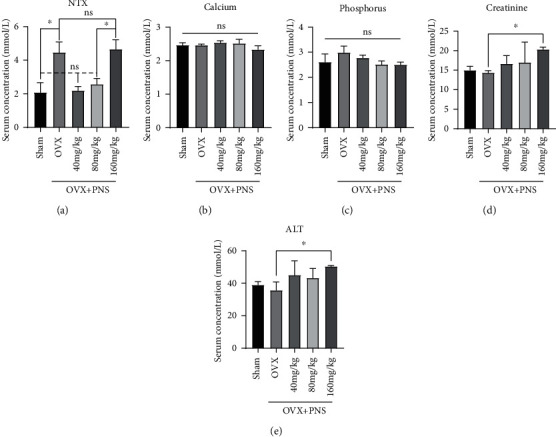
Serum biochemical quantitative analysis of N-telopeptide of (a) type I collagen (NTX), (b) serum calcium, (c) phosphorus, (d) creatinine, and (e) alanine aminotransferase (ALT) in the sham, OVX, and PNS-treated groups (“ns” represents no significant difference; ^∗^*P* < 0.05; *N* = 6).

In order to evaluate the pharmaceutic effects of PNS on bone, liver, and kidney metabolism, the serum collected from each group were tested for several indicators (Figure 2). NTX is a specific biochemical indicator of bone resorption that is generated as a result of osteoclast activity on bone. Compared with the sham operation group, the serum NTX (Figure 2(a)) of OVX group and OVX+PNS (160 mg/kg) group were significantly higher, while OVX+PNS (40 and 80 mg/kg) group showed no significant difference. These results indicated that PNS (40 and 80 mg/kg) inhibited the activities of osteoclasts in osteoporotic mice, but higher dose (160 mg/kg) of PNS intake would reverse the effect. As for the serum calcium and phosphorus (Figures 2(b) and 2(c)), there was no significant difference among the groups. The serum CREA and ALT (Figures 2(d) and 2(e)) were tested to access the function of kidney and liver, and high levels of them could reflect drug-induced liver and kidney injury. Notably, the creatinine and ALT in OVX+PNS (160 mg/kg) were significantly higher than the OVX group, and there are no significant differences among other groups. High dose (160 mg/kg) of PNS intake could cause damage to kidney and liver, which may be the reason why it had the opposite pharmacological effect compared with the lower-dose (40 and 80 mg/kg) PNS groups.

### 3.3. Micro-CT Analysis

**Figure 3:**
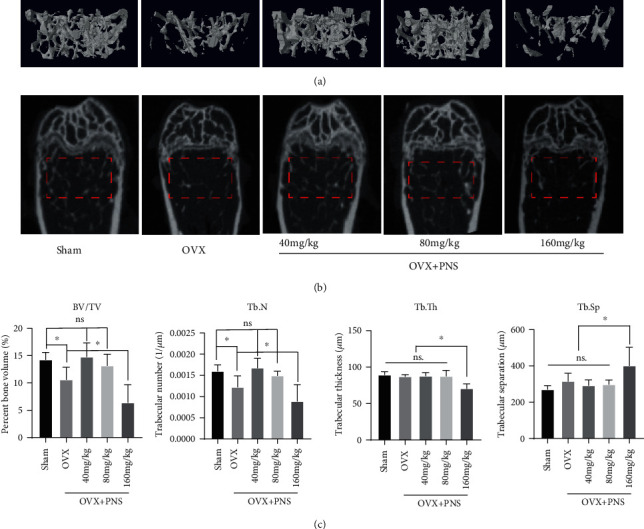
(a) Representative micro-CT 3D reconstructed images and (b) coronal images of trabecular bone in distal femur of sham, OVX, and PNS-treated groups. The red dotted line area represents the VOI for 3D reconstruction, and the quantitative analysis of the histomorphometry (c), including percent bone volume (BV/TV), trabecular thickness (Tb.Th), trabecular number (Tb.N), and trabecular separation (Tb.Sp), was measured (“ns” represents no significant difference; ^∗^*P* < 0.05; *N* = 6).

Micro-CT 3D reconstructed images and coronal images (Figure 3) of trabecular bone in distal femur showed distinct differences among sham operation, OVX, and PNS-treated groups. In general, compared with sham operation group, bone mass reduced significantly in OVX group, which means the osteoporotic mouse model was successful. When treated with 40 mg/kg or 80 mg/kg PNS, the bone mass restored significantly. However, if the concentration of PNS increased to 160 mg/kg, the antiosteoporosis effect disappeared. Specifically, in the quantitative analysis of BV/TV and Tb.N, the sham group, 40 mg/kg, and 80 mg/kg PNS group showed no significant difference (*P* > 0.05), but OVX group showed significantly decrease (*P* < 0.05), and 160 mg/kg PNS group showed even more decrease. Then, in the quantitative analysis of Tb.Th and Tb.Sp, the sham, OVX, 40 mg/kg, and 80 mg/kg PNS groups showed no significant difference (*P* > 0.05), while the 160 mg/kg PNS group significantly decreased (*P* < 0.05) in Tb.Th and increased (*P* < 0.05) in Tb.Sp.

The above results indicated that intake of appropriate dose of PNS can increase the number of trabecular bone and bone volume and in general reveal an antiosteoporotic effect; however, high dose of PNS intake would even worsen the bone loss in the osteoporotic mouse model.

### 3.4. HE Staining and Safranin O-Fast Green Staining

**Figure 4:**
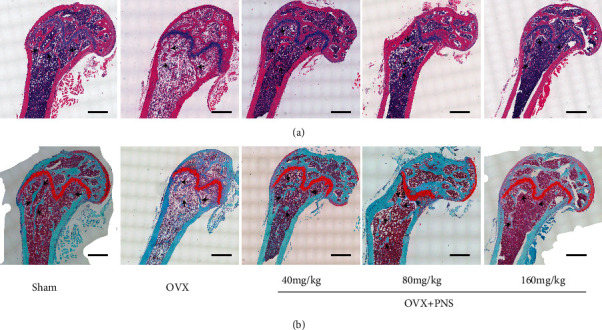
Representative sagittal images of (a) HE staining (scale bars = 500 *μ*m) and (b) Safranin O-Fast Green staining (scale bars = 500 *μ*m) of trabecular bone in distal femur of sham, OVX, and PNS-treated groups. Black arrowheads point out the typical trabecular bone in different groups.

In order to analyze the subtle changes of bone microstructure at histological and cytological levels, we performed HE staining and Safranin O-Fast Green staining among each groups (Figure 4). Bones were dyed pink in HE staining and were dyed green in Safranin O-Fast Green staining. It showed that the trabecular bone mass in the sham group and OVX+PNS (40 and 80 mg/kg) group were evidently higher than the OVX group and OVX+PNS(160 mg/kg) group, which was in consonance with the results of micro-CT morphometry.

### 3.5. Immunostaining of Osteoblasts and Vascular Endothelial Cells

**Figure 5:**
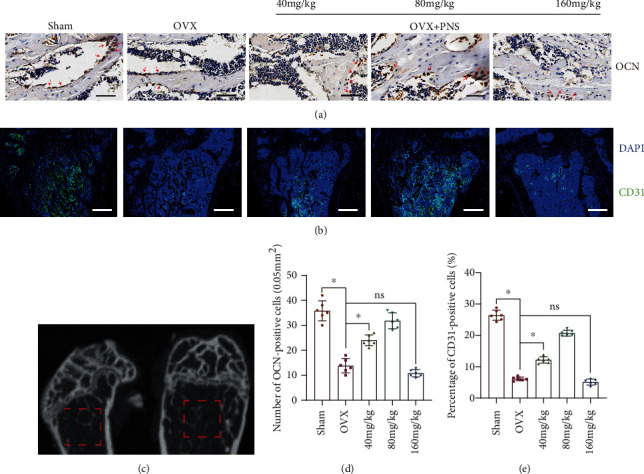
Representative images of (a) immunohistochemistry staining (scale bars = 50 *μ*m) of osteocalcin (brown) in the sham, OVX, and PNS-treated groups. Representative images of (b) immunofluorescent staining (scale bars = 200 *μ*m.) of CD31 (green) and DAPI (blue) in the sham, OVX, and PNS-treated groups. The (c) CT schematic diagram shows the approximate location of ROI (the red dotted line area) for calculating the number and percentage of OCN positive and CD31-positive cells, respectively, in each groups. Quantitative analysis of the (d) number of OCN-positive cells per 0.05 mm^2^ and the (e) percentage of CD31-positive cells was made according to the ROI (“ns” represents no significant difference; ^∗^*P* < 0.05; *N* = 6).

Osteocalcin is one of the major noncollagenous proteins of the bone matrix, which is synthesized and secreted by osteoblasts and is specific for bone. In the immunostaining of osteocalcin, OVX group showed a significantly decrease in the number of osteoblasts when compared with the sham operation group (Figures 5(a), 5(c), and 5(d)). Interestingly, intake of proper dosage of PNS (40 mg/kg and 80 mg/kg) could reverse the phenotype of osteoblast decrease in the ovariectomy-induced osteoporosis model. However, excessive intake of PNS (160 mg/kg) had no protection on the decrease of osteoblast.

CD31 is the most widely used markers of endothelial differentiation, although it is not entirely specific. In this study, we used immunofluorescent staining of CD31 to represent the vascular endothelial cells. Very similar to the immunohistochemistry results of osteocalcin, intake of proper dosage of PNS (40 mg/kg and 80 mg/kg) could reverse the decrease of the number of endothelial cells (Figures 5(b), 5(c), and 5(e)) caused by deficiency of estrogen, while excessive intake of PNS (160 mg/kg) had no such effect.

## 4. Discussion

In this study, we applied ovariectomy-induced osteoporosis model [21, 22] to verify our hypothesis that PNS could prevent osteoporosis by coactivating osteogenesis and angiogenesis in vivo. After a series of experiments, including serum biochemical analysis, micro-CT morphometry, and histological assessment, we proved that a moderate dose of PNS (40 mg/kg and 80 mg/kg) could prevent bone loss by promoting angiogenesis and osteogenesis. The decrease in ovarian estrogen secretion after menopause is the initial cause of rapid bone loss in middle aged and elderly women, with an annual bone loss rate of 3-5% within 10 years, which mainly affects the trabecular bone [23]. In our experiments, micro-CT images clearly show the trabecular bone loss in the distal femur (Figure 3) 9 weeks after the OVX operation, indicating that the OVX mouse model could perfectly simulate the rapid occurrence of postmenopausal osteoporosis in human.

In traditional Chinese medicine, PNS has vasoactive effect [24], and many studies had also proved its function in promoting angiogenesis. For instance, a study found that PNS can promote several features of angiogenesis in HUVECs in vitro and in zebrafish in vivo through the activation of the VEGF-KDR and PI3K-Akt-eNOS pathways [25], and another study confirmed the efficacy of PNS in upregulating VEGF-A, VEGFR-1, and VEGFR-2 signal systems in BMSCs [26]. It is well known that the coupling of angiogenesis and osteogenesis plays a central role in the anabolism maintaining bone homeostasis [18, 27], but no research had explored whether the antiosteoporotic effect of PNS was due to promoting angiogenesis. In this study, we had observed that appropriate dose (40 mg/kg to 80 mg/kg) of PNS intake could prevent the loss of osteoblast and vascular endothelial cell caused by estrogen deficiency at the same time, and the difference of bone mass between groups was not related to body weight, which confirmed our notion to some extent. However, high dose (160 mg/kg) of PNS intake had not only no effect in preventing bone loss but even caused more serious osteoporosis, which may be due to the damage of liver and kidney function caused by excessive dose of the PNS extract, leading to a further deterioration in bone homeostasis. During the experimental design phase, we estimated the appropriate dose in mice (40~80 mg/kg/day) according to the recommendation in the human body (150-300 mg/day), and the safety of these doses was confirmed by our study. The reason why we set up a high dose of 160 mg/kg is because we want to observe the pharmacological effects and toxicity of PNS in a high dosage, which are rarely discussed in other similar researches. Our study also indicates that when the intake of PNS was doubled from its recommended amount, it showed reverse pharmacological effects and hepatorenal toxicity.

Besides the angiogenesis promoting effect, PNS had been reported to reveal estrogen-like activities in vivo [12, 13]. Estrogen is an important regulator of osteoblast differentiation and activity, which can promote osteogenic differentiation of mesenchymal stem cell and prolong the life span of osteoblasts by inhibiting apoptosis [28]. Estrogen receptors (ER) include ER *α* and ER *β* subtypes. ER *α* mediates most of the effects of natural estrogen ligands, mainly expressed in cortical bone, while ER *β* mediates the effect of phytoestrogens on bone, mainly expressed in trabecular bone [29, 30]. PNS is one of the phytoestrogens, so we got an interesting hypothesis that PNS promotes osteogenesis and angiogenesis simultaneously or their coupling through activating ER *β* and its downstream signaling pathway in trabecular bone, which will be the next stage of our work. What is more, in the serum biochemical analysis part, the serum NTX result may indicate that PNS could have effect on osteoclasts; however, it needs more experimental evidence in the future.

Although it has been confirmed that appropriate concentration of PNS can prevent bone loss, the mechanism of the opposite pharmacological effect of high-dose PNS is not clear, and the specific mechanism of PNS promoting angiogenesis is not well explained. However, compared with the injection administration route of classic antiosteoporosis drugs, like PTH and bisphosphonate, the oral administration characteristics of PNS would make it easier for patients to take and therefore improve compliance [23, 31]. And because PNS has been used clinically for many years, it is easier to apply for clinical trials with expanded indications, making it a potential antiosteoporotic drug in the future.

## 5. Conclusions

In this study, we strongly confirmed the pharmacological effects of appropriate dose of PNS on promoting angiogenesis and preventing bone loss in a mouse osteoporotic model, which may provide a new potential treatment for the prevention of osteoporosis in postmenopausal women.

## Data Availability

All the data in this manuscript are available on request through contacting the author Hao Hu directly, and the contact email address is huhao7@mail2.sysu.edu.cn.
